# Comparative assessment of direct and indirect cold atmospheric plasma effects, based on helium and argon, on human glioblastoma: an in vitro and in vivo study

**DOI:** 10.1038/s41598-024-54070-4

**Published:** 2024-02-13

**Authors:** Mahdiyeh Bakhtiyari-Ramezani, Mojtaba Nohekhan, Mohammad Esmaeil Akbari, Fereshteh Abbasvandi, Mahdis Bayat, Atieh Akbari, Meysam Nasiri

**Affiliations:** 1 Plasma Physics and Nuclear Fusion Research School, Nuclear Science and Technology Research Institute (NSTRI), P.O. Box: 14399-53991, Tehran, Iran; 2 Cancer Research Center, Shahid Beheshti University of Medical Sciences, Tehran, Iran; 3 ATMP Department, Breast Cancer Research Center, Motamed Cancer Research Institute, ACECR, Tehran, Iran; 4 Department of Cellular and Molecular Biology, School of Biology, Damghan University, Damghan, Iran

**Keywords:** Cold atmospheric plasma jet, Glioblastoma multiform, Cancer therapy, Cancer therapy, Physics

## Abstract

Recent research has highlighted the promising potential of cold atmospheric plasma (CAP) in cancer therapy. However, variations in study outcomes are attributed to differences in CAP devices and plasma parameters, which lead to diverse compositions of plasma products, including electrons, charged particles, reactive species, UV light, and heat. This study aimed to evaluate and compare the optimal exposure time, duration, and direction-dependent cellular effects of two CAPs, based on argon and helium gases, on glioblastoma U-87 MG cancer cells and an animal model of GBM. Two plasma jets were used as low-temperature plasma sources in which helium or argon gas was ionized by high voltage (4.5 kV) and frequency (20 kHz). In vitro assessments on human GBM and normal astrocyte cell lines, using MTT assays, flow cytometry analysis, wound healing assays, and immunocytochemistry for Caspase3 and P53 proteins, demonstrated that all studied plasma jets, especially indirect argon CAP, selectively induced apoptosis, hindered tumor cell growth, and inhibited migration. These effects occurred concurrently with increased intracellular levels of reactive oxygen species and decreased total antioxidant capacity in the cells. In vivo results further supported these findings, indicating that single indirect argon and direct helium CAP therapy, equal to high dose Temozolomide treatment, induced tumor cell death in a rat model of GBM. This was concurrent with a reduction in tumor size observed through PET-CT scan imaging and a significant increase in the survival rate. Additionally, there was a decrease in GFAP protein levels, a significant GBM tumor marker, and an increase in P53 protein expression based on immunohistochemical analyses. Furthermore, Ledge beam test analysis revealed general motor function improvement after indirect argon CAP therapy, similar to Temozolomide treatment. Taken together, these results suggest that CAP therapy, using indirect argon and direct helium jets, holds great promise for clinical applications in GBM treatment.

## Introduction

Glioblastoma multiforme (GBM) is the most common aggressive primary tumor of the central nervous system (CNS) in adults. This malignancy constitutes 14.5% of all CNS tumors and accounts for 48.6% of malignant tumors within the CNS^1,2^. Individuals diagnosed with GBM confront a grim prognosis, as their median overall survival (OS) is merely 15 months^3,4^. Over the years, research has yielded numerous advancements in glioblastoma treatment. Conventional therapy for GBM relies on a combination of chemotherapy, maximal safe surgical resection, and radiotherapy; however, each component presents its own limitations^5,6^. Despite progress in glioblastoma treatment approaches, the cancer remains highly resistant due to its diverse nature and aggressiveness. Challenges include drug incapability to breach the blood–brain barrier^7^, tumor cell resistance to conventional therapies, limited brain capacity for spontaneous glioblastoma repair, and brain cell damage from traditional treatments^8,9^. Given that glioblastoma tumors typically originate deep within the brain, the quest for new treatment methods, especially non-invasive ones, becomes imperative. Such methods aim to enhance anti-cancer efficacy while sparing normal tissues from harm^10,11^.

Recent developments in physics research have led to the creation of cold atmospheric pressure plasma (CAP), a type of plasma generated at relatively "cold" temperatures, including room temperature. Several gases, such as Helium, Argon, Nitrogen, Heliox, and air, can be utilized to generate CAP^11,12^. CAP consists of a partially ionized gas containing a reactive mix of positively and negatively charged ions, along with reactive species like OH, O, H2O2, O3, NO, NO2, and more, as well as neutral particles, electromagnetic waves, and UV radiation. These combined components are collectively referred to as reactive agents (RAs)^13^. Plasma's ability to generate a diverse array of RAs makes it valuable in various fields, encompassing wound healing, medical applications, dentistry, sterilization, dermatology, and clinical oncology^14^.

Recently, CAP has consistently demonstrated positive anticancer activity when used as a standalone therapy. Various mammalian cell types, including neuroblastoma^15^, pancreatic carcinoma^16,17^, colon carcinoma^18^, skin carcinoma^19^, hepatocellular carcinoma^20^, cervical carcinoma^21^, lymphoblastic leukemia^22^, lung carcinoma^23^, and breast cancer^24^, have been employed for both in vitro and in vivo plasma therapy. To date, all reported anti-cancer effects of cold atmospheric pressure (CAP) treatment, both in vitro and in vivo, have generally been attributed to the cellular responses induced by CAP-generated reactive oxygen and nitrogen species (RONS) and free radicals. These entities are recognized for their toxicity to cancer cells, ultimately leading to the induction of apoptotic cell death^25,26^.

Initial observations introduced CAP as a potentially useful ablative therapy in the treatment of glioblastoma^27,28^. Our previous study also exhibit that plasma treatment can be used as an effective and efficient method to treat the GBM cancer^29^. Based on these preclinical studies, CAP has holds great promise for clinical applications in GBM treatment^5,10,27,30–34^. However, these studies are still in the preclinical phase, and determining the optimal dose and duration of CAP treatment is necessary for complete GBM cure. The present study for the first time, aimed to assess and compare dose, time, and direction-dependent cellular effects of two CAPs, based on argon and helium gases, on glioblastoma U-87 MG cancer cells and an animal model of GBM. We also examined the possible cellular and molecular mechanisms involved in this plasma therapy.

## Results

### In vitro results

#### Assessing optical emission spectroscopy from cold atmospheric plasma jets

**Figure 1:**
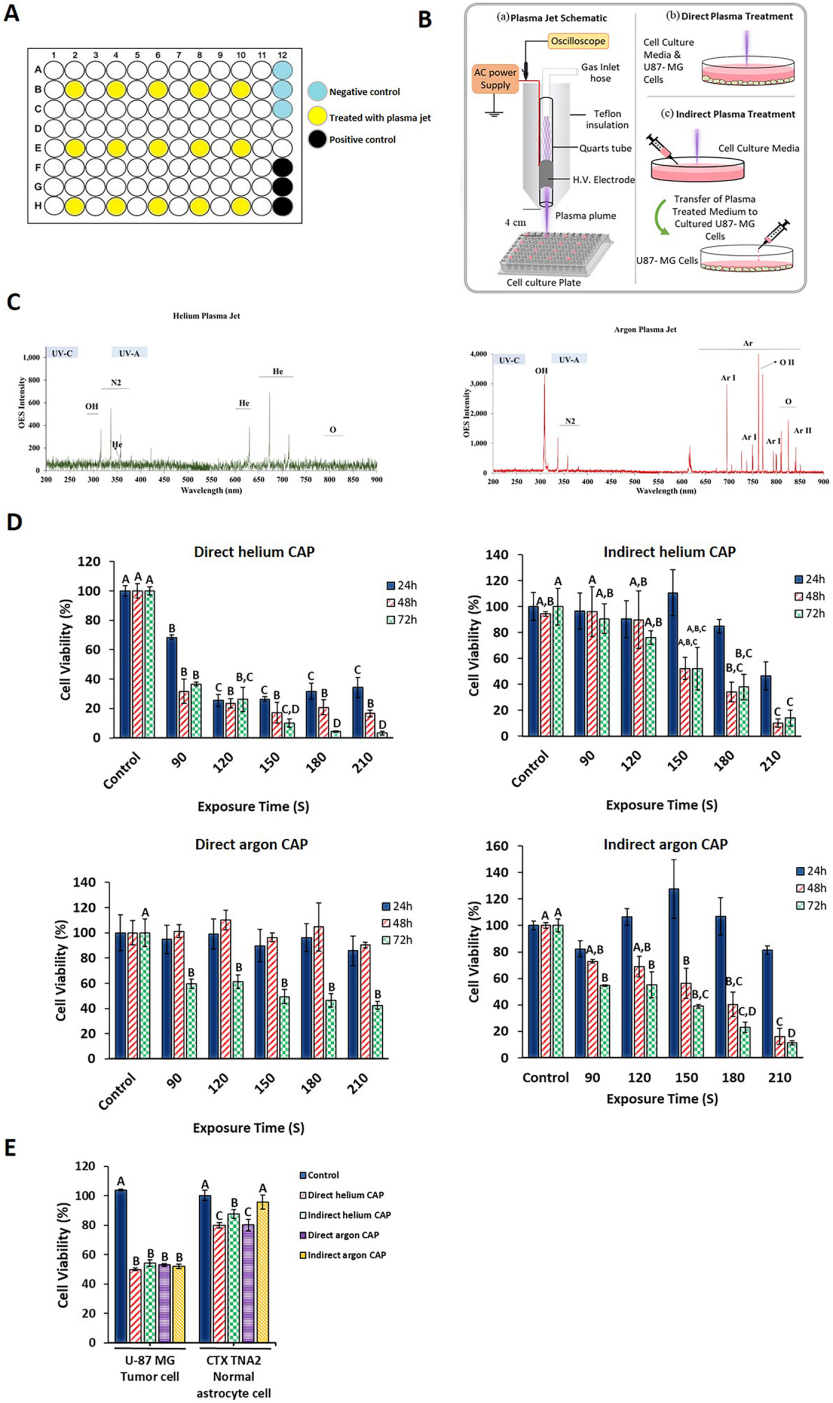
(**A**) the seeding format of the cells in the 96-well plate. The yellow wells indicate the cells treated with plasma at different times, the black wells indicate the positive control, and the blue wells indicate the negative control. (**B**) The Plasma Jet System. Schematic of the direct and indirect plasma jets interacting with U-87 MG cells. (**C**) Optical Emission Spectra of Cold Atmospheric Argon Plasma Jet (red line) and Helium Plasma Jet (green line). Using OPTC spectroscopy, optical emission spectroscopy detected nitrogen, oxygen, and hydroxyl peaks in both helium and argon CAP jets. However, the line intensity was notably higher in the argon plasma jet. (**D**) Relative viability of U-87 MG cells after treatment for 90, 120, 150, 180, and 210 s with different gas plasmas and 24, 48, and 72 h of culture after treatment with plasma. All data represent the mean ± SEM, and all experiments were performed in triplets. (**E**) Treatment of U-87 MG tumor cells and CTX TNA2 normal astrocyte cells with IC50 dose of helium and argon plasmas directly and indirectly. Data were analyzed using One-way ANOVA with Bonferroni multiple comparison post hoc test. Columns that have no letter in common with each other are meaningful (*P* < 0.05).

Figure 1C illustrates the emission spectra of the cold atmospheric helium and argon plasma jets. As shown, atomic lines of argon (lines between 680 to 850 nm), hydroxyl radicals (line at 308.9 nm), nitrogen molecules (lines at 337.13 nm, 357.69 nm, 380.49 nm), and atomic oxygen (at 762.7 nm) can be observed. The spectral range in which helium peaks are usually obtained is from 600 to 750 nm. Moreover, the OH and O lines are detected for cold atmospheric helium and argon plasma jets. However, the line intensity was higher in the cold atmospheric argon plasma jet.

#### The growth-inhibitory effects of various cold atmospheric plasmas on U-87 MG tumor cells and CTX TNA2 normal astrocyte cells

To determine the optimal exposure time for direct and indirect helium and argon plasma treatments, we assessed the viability of U-87 MG cells at five different exposure times: 90, 120, 150, 180, and 210 s. We conducted the assessment after 24, 48, and 72 h of continuous cultivation following the exposure using the MTT assay. Our findings revealed that the rate of cell death is influenced by both the exposure time and the duration of cultivation after treatment. Based on the calculated IC50 values, the optimal exposure times were found to be 70.15 and 138 s at the 48-h post-treatment time point for direct and indirect helium CAPs, respectively, and 202 and 116 s at the 72-h post-treatment cultivation time point for direct and indirect argon CAPs, respectively (See supplementary table S2 and Fig. 1D). Since the IC50 value for direct helium plasma was obtained after 48 h of treatment (15.41 s), which falls outside the range of plasma treatment times, we conducted additional MTT tests with exposure times of 30, 45, 60, 75, and 90 s after 48 h of continuous cultivation. The purpose was to calculate the IC50 of direct helium plasma more accurately. The results revealed that the IC50 value for pure helium plasma jet after 48 h of cultivation is 70.15 s (See supplementary figure S1).

The impact of the IC50 dose of the investigated gas plasmas on CTX TNA2 cells was also assessed. According to the results of the MTT assay, the cell viability of CTX TNA2 cells was found to be 79.88% for direct helium CAP (*P* < 0.0005), 87.68% for indirect helium CAP (*P* = 0.001), 80.17% for direct argon CAP (*P* < 0.0005), and 95.75% for indirect argon CAP (*P* = 0.44) plasma groups, respectively, when compared to untreated normal cells (Fig. 1E). These data clearly show that indirect argon CAP, while effective on U-87 MG tumor cells at IC50 dose, is safe for normal astrocyte cells and has more selectivity than other CAPs. Based on these results, in all subsequent experiments in this study, the IC50 exposure time and the cultivation times 48 and 72 h were used for helium and argon plasma, respectively.

#### Assessment of early and late apoptosis and necrosis in U-87 MG cells exposed to various CAPs

**Figure 2:**
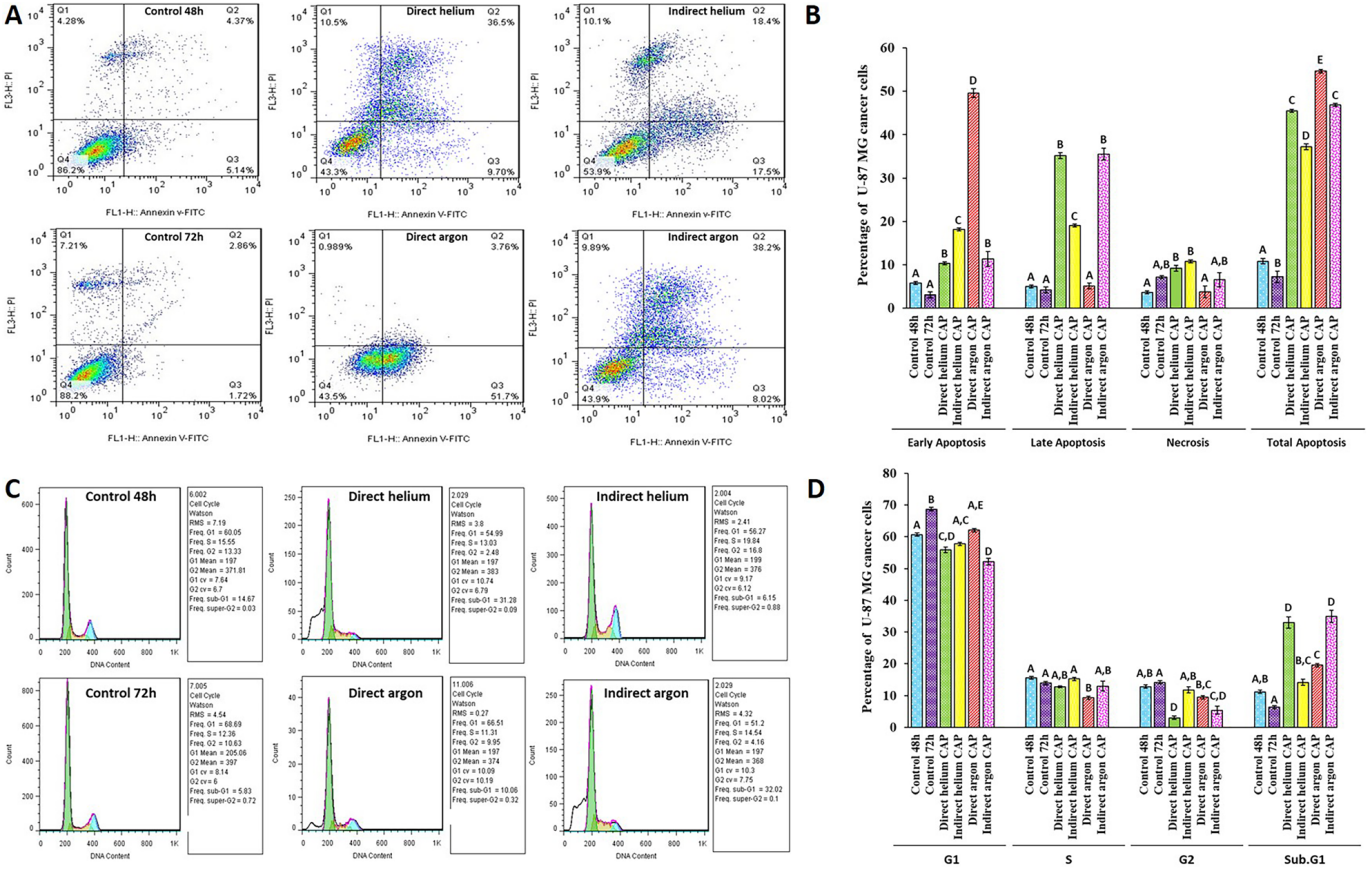
(**A**) Flow cytometry analysis using the Annexin V/PI staining method. Quadrant (Q4) represents living cells, Quadrant (Q3) represents early apoptotic cells, Quadrant (Q2) represents late apoptotic cells, and Quadrant (Q1) represents necrotic cells. (**B**) Quantitative plot of flow cytometry data. Comparing the rate of apoptosis and necrosis of U-87 MG cells due to treatment with different plasma jets. (**C**) Assessment of cell cycle distribution in response to various plasma jet treatments. (**D**) A quantitative comparison of the effect of different plasma jets on the proportion of cell cycle arrest in U-87 MG cells. All data represent the mean ± SEM, and all experiments were performed in triplets. Data were analyzed using One-way ANOVA with Bonferroni multiple comparison post hoc test. Columns that have no letter in common with each other are meaningful (*P* < 0.05).

Early apoptosis, late apoptosis, and necrosis were assessed using the Annexin V/Propidium Iodide staining method and analyzed by flow cytometry. The results showed that all studied plasmas significantly increased the induction of apoptosis in U-87 MG cells compared to the control group (*P* < 0.001) (Fig. 2A, B). The interesting point is that direct argon CAP induced more early apoptosis, while direct and indirect helium CAPs, as well as indirect argon CAP, induced more late apoptosis compared to the controls (Fig. 2A, B). In addition, the rate of necrotic death did not change significantly in the direct argon CAP and indirect argon CAP groups compared to the control group (Fig. 2A, B).

Since an accelerated cell cycle indicates increased proliferation, we investigated how treatment with different plasmas affects cell cycle progression in U-87 MG cells. The cell cycle analysis demonstrated that the treatment of U-87 MG cells with direct helium, direct argon, and indirect argon CAPs resulted in a significant decrease in the number of cells in the G2 phases of the cell cycle, indicating enhanced growth inhibition compared to the control group (*P* < 0.001) (Fig. 2C, D). One of the primary features of apoptosis is the fragmentation of internucleosomal DNA. Apoptotic cells in DNA content histograms can be identified as those with fractional DNA content, often referred to as sub-G1 cells. In our experiment, we observed an increase in cell death when cells were exposed to direct helium, direct argon, and indirect argon CAPs (Fig. 2C, D). This increase in cell death was correlated with a rise in the percentage of cells in the sub-G1 phase. Meanwhile, only direct argon CAP resulted in a significant reduction in the number of S-phase cells compared to the control group (Fig. 2C, D).

#### The effect of direct and indirect plasmas of helium and argon on the migration of U-87 MG cells

**Figure 3:**
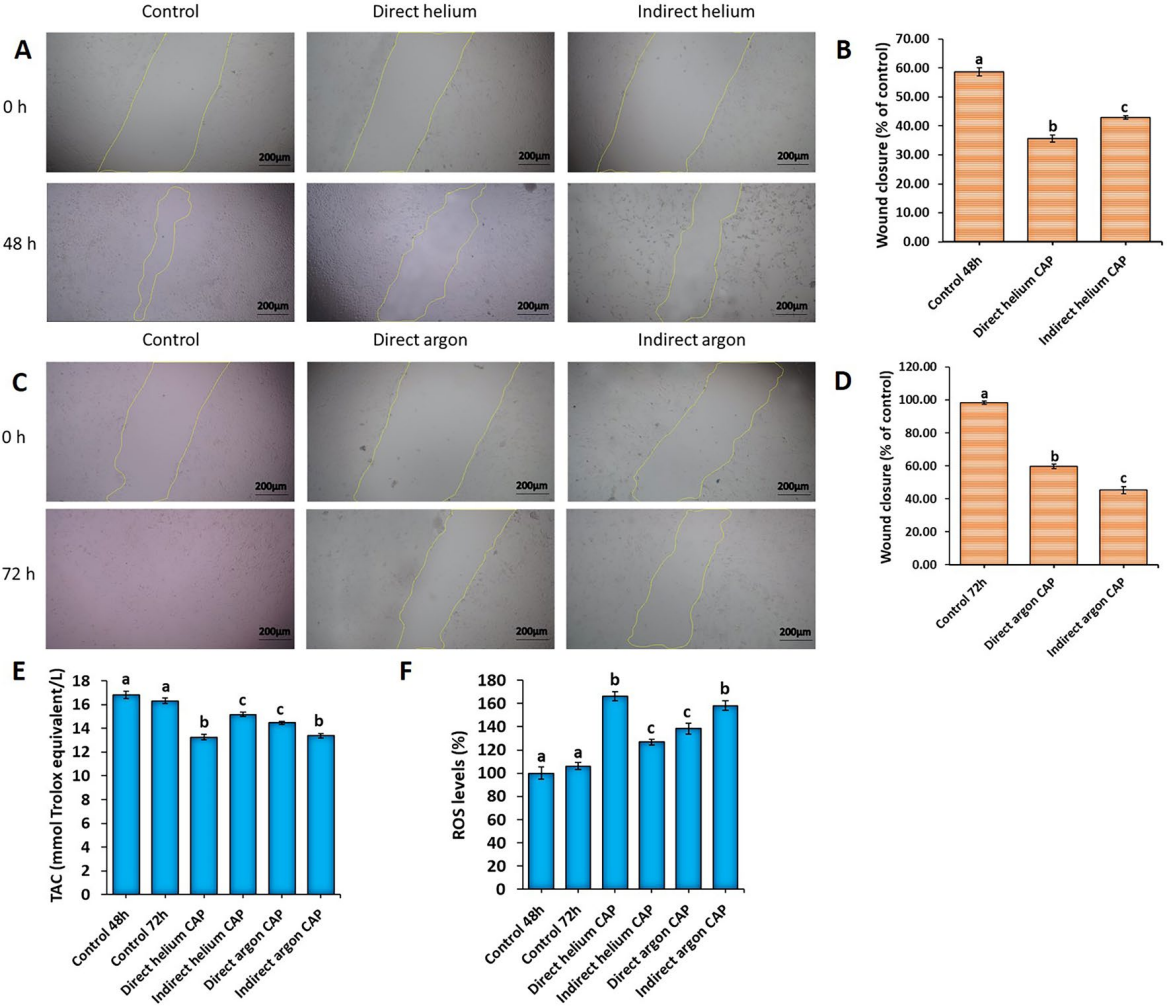
(**A**, **C**) Microscopic images depicting wound healing experiments involving various plasma jets (The scale bar is 200 µm). (**B**, **D**) Quantitative comparison of the effect of different plasma jets on the migration rate of U-87 MG cells. (**E**, **F**) Evaluation of the total antioxidant capacity (TAC) and reactive oxygen species (ROS) levels in U87-MG cells exposed to various CAPs. All data represent the mean ± SEM, and all experiments were performed in triplets. Data were analyzed using One-way ANOVA with Bonferroni multiple comparison post hoc test. Columns that have no letter in common with each other are meaningful (*P* < 0.05).

We conducted a scratch wound healing assay to evaluate the impact of different CAPs on cell migration. After 48 h of treatment, the results demonstrated that direct helium and indirect helium CAPs treatment led to 35.57% and 42.86% wound closure, respectively, compared to the control group (58.56%) (*P* < 0.001) (Fig. 3A, B). In the case of argon plasma treatment, the wound closure was 59.74% for direct irradiation (*P* < 0.001) and 45.26% for indirect irradiation after 72 h of cultivation (*P* < 0.001) compared to the control group (98.23%) (Fig. 3C, D). These findings provide clear evidence that direct helium and indirect argon CAPs exhibit the highest inhibition of cell migration among the studied gas plasmas. Moreover, the rate of inhibition of cell migration compared to the control was higher in indirect argon plasma than in direct helium CAP.

#### Assessment of total antioxidant capacity and levels of reactive oxygen species in U87-MG cells subjected to various CAP treatments

In the next step, to investigate the possible mechanism of the effect of studied plasmas on U-87 MG cells, the levels of ROS and total antioxidant capacity (TAC) in these cells were evaluated. The results showed that the greatest decrease in TAC level occurred in the direct helium CAP (13.24 ± 0.24) and indirect argon CAP (13.38 ± 0.17) groups, compared to the control group (16.81 ± 0.33) (*P* < 0.001, *P* < 0.001 respectively) (Fig. 3E). Additionally, there was no significant difference between direct helium and indirect argon CAPs (*P* = 1) (Fig. 3E). The highest amount of ROS production compared to the control group (100 ± 5.15) was also obtained in the direct helium CAP (166 ± 3.79) and indirect argon CAP (158 ± 4.12) groups (*P* < 0.001, *P* < 0.001 respectively) (Fig. 3F). Although there was no significant difference between these two groups (P = 1) (Fig. 3F).

#### Utilizing immunocytochemistry to assess the expression of P53 and caspase3 proteins in U87- MG cells exposed to various plasma treatments

**Figure 4:**
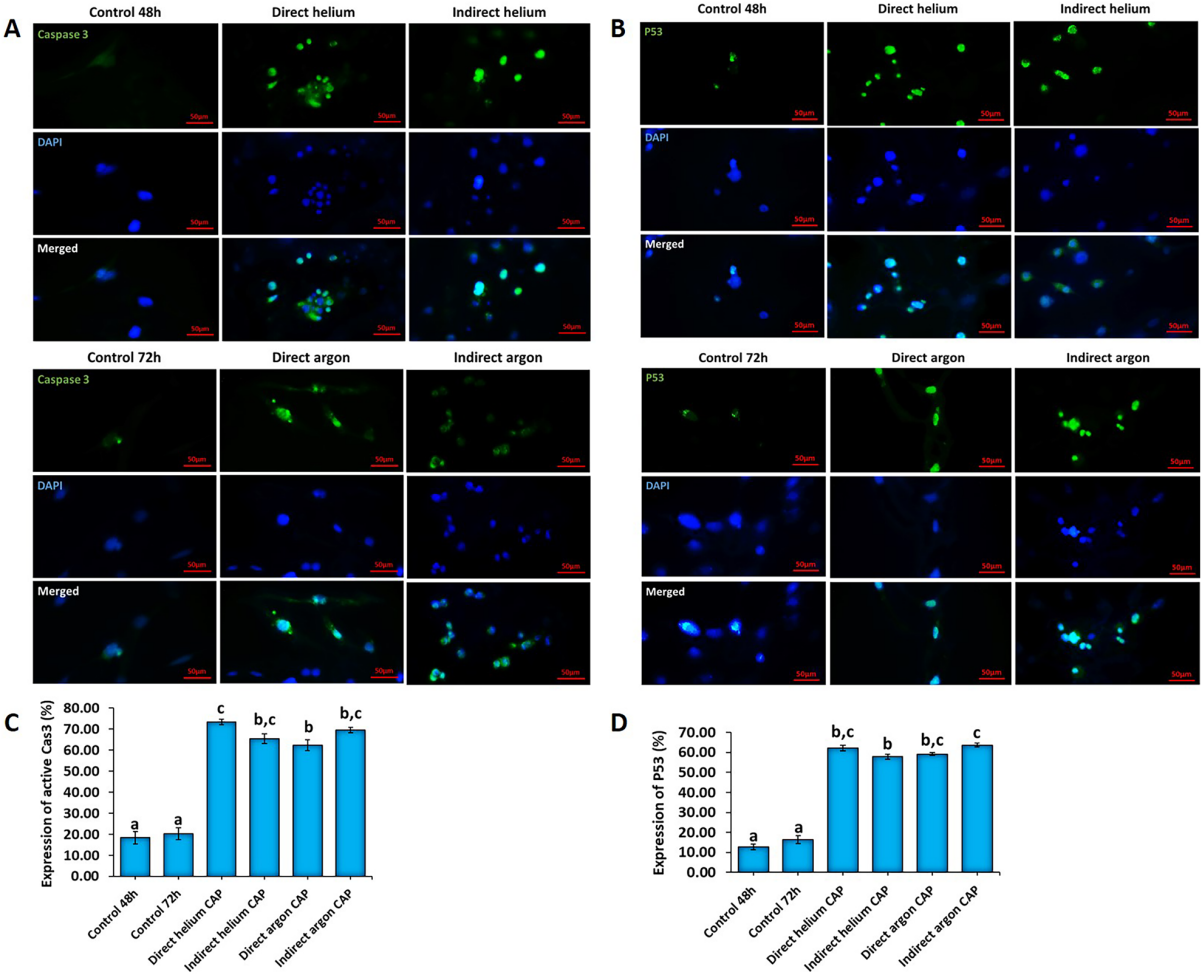
(**A**, **B**) Immunocytochemistry photomicrographs depicted the effect of direct and indirect helium and argon CAPs on the expression of P53 and Caspase-3 in U87-MG cells (the scale bar is 50 µm). (**C**, **D**) Quantitative comparison of immunocytochemical results of the effect of different plasma jets on the expression of P53 and Caspase3 proteins in cells. All data represent the mean ± SE, and all experiments were performed in triplets. Data were analyzed using One-way ANOVA with Bonferroni multiple comparison post hoc test. Columns that have no letter in common with each other are meaningful (*P* < 0.05).

In the following step, we examined the impact of direct and indirect CAPs of helium and argon on the expression of two critical factors: P53 and Caspase-3 proteins (Fig. 4A-D). These factors play essential roles in inhibiting the proliferation, angiogenesis, migration, and escape from apoptosis of glioblastoma tumor cells^35,36^. The results from immunocytochemistry (ICC) revealed a significant increase in the expression levels of Caspase3 and P53 proteins in all studied groups compared to the control group (*P* < 0.001) (Fig. 4A–D). Moreover, the highest expression levels of Caspase3 and P53 were observed in the direct helium CAP (73.4 ± 1.25 and 62.21 ± 1.47, respectively) and indirect argon CAP (69.6 ± 1.28 and 63.69 ± 0.92, respectively) groups compared to the control group (18.34 ± 2.93 and 12.78 ± 1.14, respectively) (*P* < 0.05) (Fig. 4A–D). Interestingly, there was no significant difference in expression levels between the direct helium and indirect argon CAPs groups (*P* = 1) (Fig. 4A–D).

### In vivo results

#### Comparing the therapeutic effects of various atmospheric cold plasmas among themselves and with temozolomide

**Figure 5:**
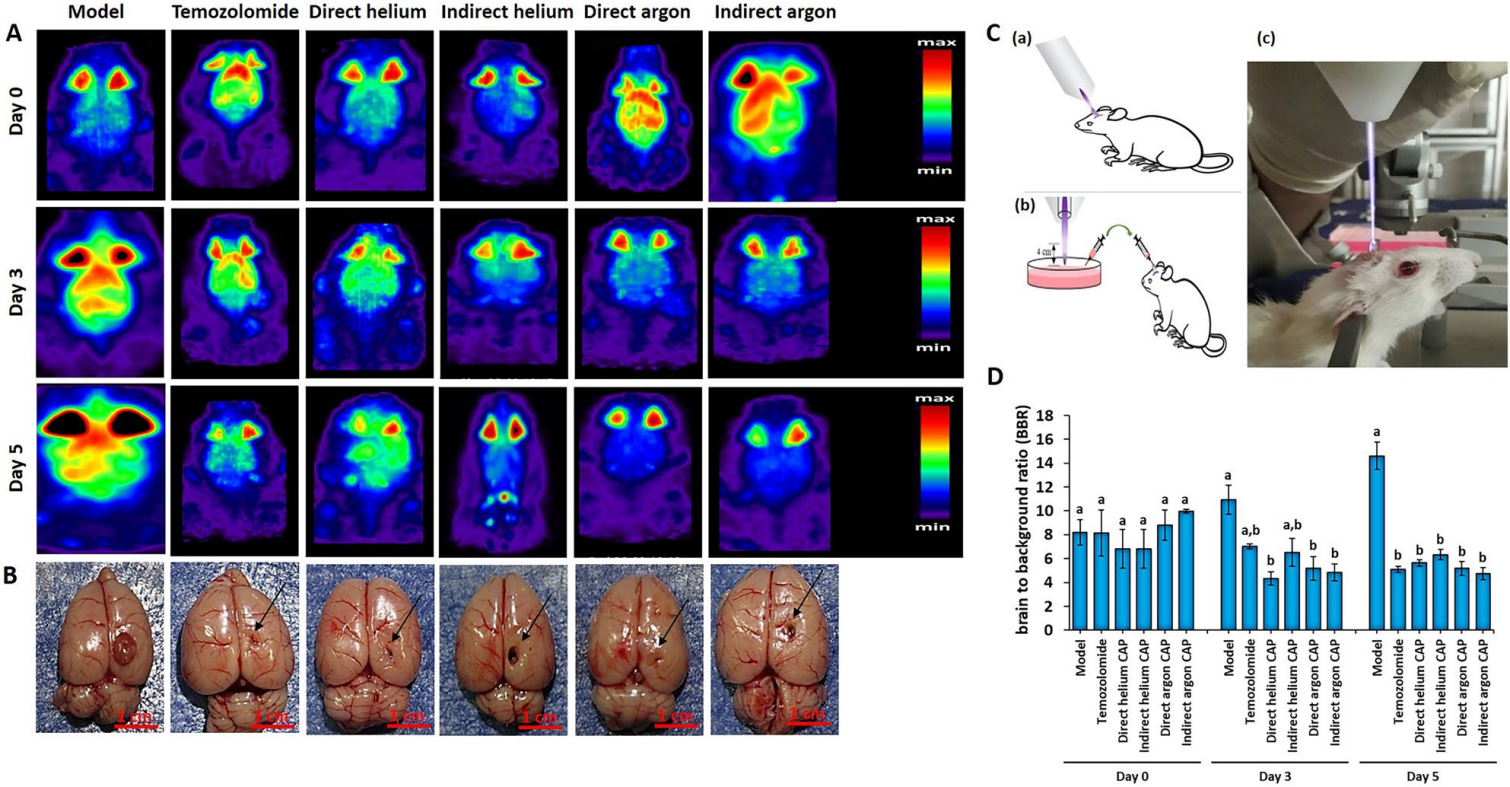
PET scans depicting images at the initial measurement (day 0), as well as at 3 and 5 days after either a single treatment with CAPs or the administration of TMZ. (**B**) Macroscopic representation of reduced tumor volume in the brain of animals treated with temozolomide and different plasma jets. (**C**) Schematic representation of the (a, c) direct and (b) indirect CAPs treatment for GBM in vivo. (**D**) Quantitative comparison of PET imaging data between different studied groups. Data were analyzed using One-way ANOVA with Bonferroni multiple comparison post hoc test. Columns that have no letter in common with each other are meaningful (*P* < 0.05).

Based on our in vitro findings, which showed that CAP jet has the ability to induce apoptosis, inhibit the proliferation and migration of U-87 MG cells, we then investigated it in an animal model of GBM. In vivo PET imaging showed marked and progressive tumor growth over 5 days in model group; thus, the tumor size in this group increased by 33.3 ± 14.01% and 78.4 ± 7.47% on days 2 and 5, respectively (Fig. 5A, D). Temozolomide treatment did not prevent tumor progression at 48 h after treatment. Meanwhile, one treatment dose with direct helium and direct and indirect argon plasmas significantly reduced the tumor size compared to the model group. In addition, 5 days after treatment, temozolomide along with all studied plasmas significantly reduced tumor growth (Fig. 5A, D). It was interesting to note that there was no significant difference between the temozolomide groups and the studied plasmas on day 5 (Fig. 5A, D). After removing the brain tissue, the tested samples were examined macroscopically in terms of tumor volume. As shown in Fig. 5B, the largest tumor volume was observed in the model group, and in other groups, the tumor volume was visibly reduced.

**Figure 6:**
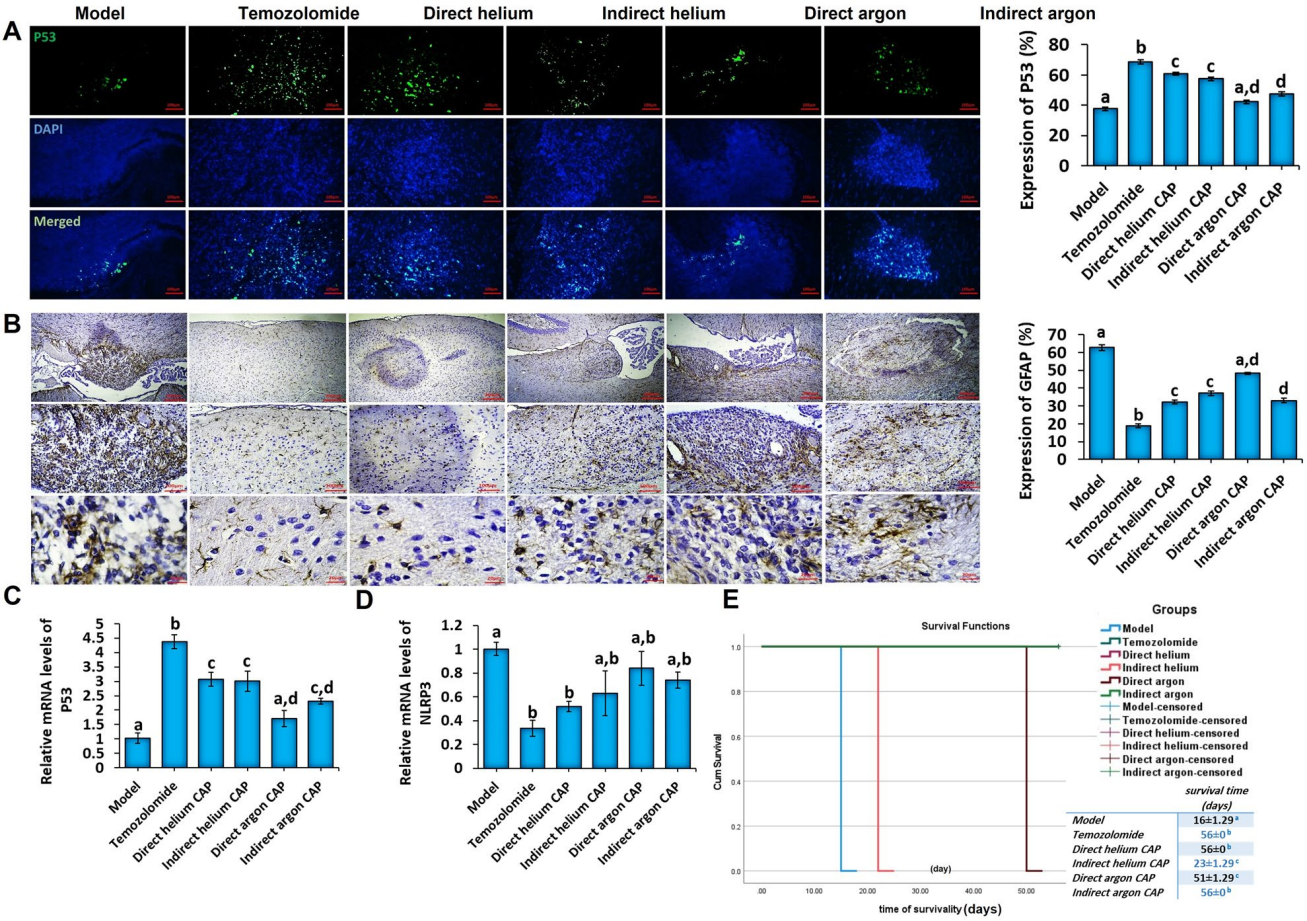
(**A**) Representative Immunohistochemical of P53 expression (left), Quantitative comparison of immunohistochemical results of p53 expression (right) (The scale bar is 100 µm), (**B**) Representative Immunohistochemical of GFAP expression (left), Quantitative comparison of immunohistochemical results of GFAP expression (right) (The scales bar from top row to bottom are 200 µm, 100 µm, and 20 µm). (**C**, **D**) Comparison of the relative mRNA expression of P53 (left) and NLRP3 genes (right) in the brain of animals treated with different gas plasmas and temozolomide. (**E**, **F**) Kaplan–Meier diagram to analyze the difference in median survival of mice treated with different plasmas and temozolomide 8 weeks after treatment.

Next, in order to compare the inhibitory effect of the studied CAP jets with temozolomide, on inhibiting the growth and proliferation of GBM tumor cells, the expression level of P53 protein was investigated. As can be seen in Fig. 6A, by applying temozolomide and direct and indirect helium CAPs and indirect argon CAP treatment, the expression of P53 gene showed a significant increase compared to the model group. The qPCR data also confirm the changes in P53 gene expression in the studied groups (Fig. 6C).

The intermediate filament (IF) protein glial fibrillary acid protein (GFAP) is a signature type III IF protein of glioma cells that has been implicated in tumor migration. While further clarification is needed regarding the relationship between GFAP and GBM metastasis, the staining of GFAP is acknowledged as a standard diagnostic indicator for GBM when examining samples obtained from within the central nervous system^37,38^. The results obtained from the fluorescent microscope images showed that the level of GFAP protein expression in cells treated with temozolomide, helium with direct and indirect radiation and indirect argon CAP has decreased significantly compared to the model group (Fig. 6B).

Nod-like receptor (NLR) family pyrin domain containing 3 (NLRP3) plays a critical role as a vital constituent within the NLRP3 inflammasome, a complex comprising multiple proteins capable of triggering an inflammatory response mediated by caspase 1/interleukin-1 (IL-1) upon activation^39^. This activation is implicated in a range of inflammatory diseases and cancer pathogenesis. In the context of glioma, suppressing NLRP3 led to a decrease in glioma cell growth and invasion. Conversely, augmenting NLRP3 expression promoted cell growth^39^. qPCR results for NLRP gene showed that temozolomide and direct helium CAP significantly decreased the mRNA expression of this gene compared to the model group (Fig. 6D).

#### Comparison of the effects of plasma jets and temozolomide on the behavioral characteristics of animals

All animals in the model group died in the third week of the study. As shown in Fig. 6E, Kaplan–Meier analysis showed that temozolomide, direct helium and indirect argon CAPs treatments can significantly increase the median survival of animals (Log Rank Mantel-Cox *P* < 0.001). In the indirect helium CAP and direct argon CAP groups, the animals died in the fourth and eighth weeks, respectively (Fig. 6E).

During the study, the weight of the animals was also controlled. The temozolomide group experienced significant weight loss from week 1 to week 8 of the study (F = 5.32, *P* = 0.024) (ηp^2^ = 0.4) (Table 1). In the direct helium CAP group, there was a trend of weight loss, but it was not statistically significant (F = 3.15, *P* = 0.1) (ηp^2^ = 0.28) (Table 1). This is while the indirect argon CAP group had an increase in weight from week 1 to week 8 of the study, but it was not statistically significant (F = 3.32, *P* = 0.1) (ηp^2^ = 0.29). In addition, the trend of weight changes during 8 weeks showed a significant difference between the three groups of temozolomide, direct helium and indirect argon CAPs (F = 3.55, *P* = 0.012) (ηp^2^ = 0.23) (Table 1).

**Table 1:** The average weight (gr) of animals during 8 weeks in different studied groups.

*Group*	Week1	Week2	Week3	Week4	Week5	Week6	Week7	Week8
Model	284 ± 4	283 ± 3	–	–	–	–	–	–
Temozolomide	291.3 ± 1.5	292 ± 2.64	291 ± 1	292.3 ± 2.5	291 ± 1	284 ± 4	282.6 ± 2.5	283 ± 3
Direct helium CAP	274.6 ± 4.5	273.6 ± 3.5	272.3 ± 2.5	271 ± 1	272.6 ± 2.5	264 ± 4	272.3 ± 2.5	271 ± 1
Indirect helium CAP	252.3 ± 2.5	252.3 ± 2.5	272.6 ± 2.5	–	–	–	–	–
Direct argon CAP	294.6 ± 4.5	294 ± 3.6	294 ± 4	292.3 ± 2.5	293.6 ± 3.5	302.3 ± 2.5	301 ± 1	–
Indirect argon CAP	294 ± 4	293 ± 3	292.3 ± 2.5	294 ± 2.5	302.3 ± 2.5	301 ± 1	302.3 ± 2.5	302.3 ± 1.5

The reliable evaluation of hindlimb functioning following unilateral brain damage has been accomplished through the utilization of the ledge beam-walking test. This assessment method, serves as a locomotor test specifically designed to identify hindlimb placement dysfunction^40^. This valuable test aids in the detection of hindlimb impairments resulting from unilateral brain damage, providing crucial insights into the effects of such damage on locomotion and motor coordination. Repeated measure analysis for Ledged beam test results showed that treatment with temozolomide (F = 5.32, *P* = 0.014) (ηp^2^ = 0.26) and indirect argon plasma (F = 5.82, *P* = 0.013) (ηp^2^ = 0.28) significantly improves the general motor performance and movement balance of animals from the fourth to the eighth week (Table 2). Meanwhile, in the direct helium CAP group where the animals survived until the end of the studied time point, no significant improvement was observed in the results of the beam test (F = 1.89, *P* = 0.17) (ηp^2^ = 0.11) (Table 2). In addition, no significant differences were observed in the results of this test between the studied groups (F = 1.77, *P* = 0.11) (ηp^2^ = 0.073) (Table 2).

**Table 2:** The walking scores for ledged beam test in different groups.

	Week1	Week2	Week3	Week4	Week5	Week6	Week7	Week8
Model	1.37 ± 0.25	1.37 ± 0.25	–	–	–	–	–	–
Temozolomide	1.37 ± 0.41	1.75 ± 0.29	1.62 ± 0.48	1.75 ± 0.50	1.87 ± 0.25	1.75 ± 0.29	1.87 ± 0.25	2 ± 0
Direct helium CAP	1.62 ± 0.48	1.62 ± 0.25	1.75 ± 0.5	1.87 ± 0.25	1.87 ± 0.25	1.87 ± 0.25	1.87 ± 0.25	1.75 ± 0.5
Indirect helium CAP	1.37 ± 0.25	1 ± 0	1.75 ± 0.5	–	–	–	–	–
Direct argon CAP	1.5 ± 0.41	1.62 ± 0.48	2 ± 0	2 ± 0	1.87 ± 0.25	2 ± 0.25	2 ± 0	-
Indirect argon CAP	1.5 ± 0.41	1.75 ± 0.29	2 ± 0.25	1.87 ± 0.25	1.87 ± 0.25	2 ± 0	1.75 ± 0.5	2 ± 0

## Discussion

GBM is one of the most common cancers in the CNS, and patient prognosis is often poor^41^. A combination of maximal safe surgical resection with chemotherapy and radiotherapy has been the mainstay of GBM treatment for decades^5,6^. Complete tumor resection is virtually impossible because any damage to the brain tissue during the surgery can cause major neurological complications, which can result in reductions in overall survival^42^. Another major challenge that must be addressed for the successful treatment of GBM is drug penetration to the tumor site, its aggressive nature, and resistance to drug treatment^5^. Hence, novel methods and therapeutic targets are necessary to be identified to effectively improve survival outcomes in GBM patients^27,43^.

One promising advancement is the development of CAP technology, which exhibits specificity for in vitro and in vivo treatment of various cancers, such as glioblastoma, and has led to the establishment of a new field in medicine called "plasma oncology"^23,30,44^.

Preclinical observations have introduced CAP as a potentially useful ablative therapy in the treatment of glioblastoma^27,32^. In accordance with the literature, our previous study also demonstrates that plasma therapy can be used as an effective and efficient method to treat GBM cancer^32^. Based on these preclinical results, CAP holds great promise for clinical applications in GBM treatment^5,10,27,30^. Nevertheless, studies up to now are still in the preliminary phase, and the specific parameters of CAP therapy for a complete cure of GBM are yet to be elucidated. The current study aims to assess and compare the exposure time, duration, and direct or indirect-dependent effects of two CAPs, based on argon and helium gases, on glioblastoma U-87 MG cancer cells and an animal model of GBM.

Based on the calculated IC50 values, the optimal exposure times were 70.15 and 138 s at the 48-h post-treatment time point for direct and indirect helium CAPs, and 202 and 116 s at the 72-h post-treatment time point for direct and indirect argon CAPs, respectively. This confirms previous studies^5,34^, which revealed that the effect of CAP on cell death is influenced by the exposure time and the duration of cultivation after treatment.

As we know one of the challenges in introducing a new therapeutic approach is proving its safety for healthy cells. It is necessary to develop therapies that specifically minimize damage to healthy tissues while effectively destroying tumor cells^10,11^. Our results showed that among all CAP-treated U-87 MG cell lines, only indirect argon CAP had a selective killing effect on GBM cell lines at its IC50 dose, providing greater safety for the normal brain cells. Confirming our results, many studies have also emphasized that CAP does not harm normal tissues when applied at appropriate dosages, introducing CAP as a selective treatment modality for killing cancer cells^45,46^.

Although the exact mechanisms of CAP’s oncological advantage are not fully understood. the majority of researchers confirm that primary action of CAP is through the production of long-lived molecules such as reactive oxygen and nitrogen species (RONS) in atmospheric air or solution^47–50^. Therefore, targeting oxidative equilibrium of tumor cells is currently a recognized approach to kill cancer cells^51,52^. Consistent with the literature, our results also revealed that following CAP therapy, intracellular ROS (Reactive Oxygen Species) levels significantly increased, while TAC (Total Antioxidant Capacity) levels decreased significantly. The highest levels of ROS and the lowest levels of TAC were reported in direct helium and indirect argon CAPs treated U87MG cell lines. It is known that the selectivity of CAP against cancer cells depends on the different basal intracellular ROS levels between cancer and normal cells. Cancer cells tend to possess higher metabolism and basal ROS levels than normal cells, making them more susceptible to exogenous ROS stress, resulting promote several aspects of cancer cell proliferation and metastasis cell death^53^.

In order to find out the possible underlying effect of CAP-generated RONS, we evaluated the expression of two main genes involved in cell cycle arrest and apoptosis, p53 and caspase 3, in CAP-treated U87-MG human glioblastoma cell lines. Our results revealed that the expression of P53 and caspase-3 dramatically increased following CAP therapy, especially in direct helium and indirect argon CAPs-treated U-87 MG cells. In confirmation of the gene expression results, our flow cytometry data also showed an increase in total apoptosis and a reduction in the proportion of cells in the S and G2 phases, confirming CAP-mediated tumor growth inhibition. It has been known that dysregulation of several genes involved in apoptosis and cell proliferation, such as caspase-3 and P53, plays an important role in the pathogenesis and progression of many cancers such as GBM^24,54–56^. Previous studies stated that The ROS produced by CAP may be the main underlying mechanism of CAP-induced apoptosis^57^. The ROS targets molecular signaling pathways which ultimately led to apoptosis^49^. In these regard, some studies confirmed that CAP- generated RONS can trigger cell signaling pathways involving JNK and p38^49^ and p53^58^ which leading to promoting activation of caspases and apoptosis^49,59^. Hence, the overexpression of p53 and caspase 3 can be considered as one of the targets of ROS-induced apoptosis.

Based on our in vitro findings demonstrated that CAPs jet has the ability to inhibit the proliferation and migration and induced apoptosis of U-87 MG cells, in the next part of the study, we investigated the effect of our plasma device on animal model of glioblastoma tumors in vivo in compared with common treatment (temozolomide).

In line with our in vitro findings, we found that a single CAPs treatment was effective in partially preventing brain tumor growth. There was a significant decrease in the tumor volume of the direct helium, direct argon, and indirect argon CAPs-treated groups compared with the baseline volume over the course of the study, while the effect of temozolomide on the reduction of tumor volume was observed later on the 5th day after treatment. These striking findings demonstrate the potential of CAP alone to inhibit glioblastoma tumor growth in vivo. In confirmation of the current data, some previous studies also reported that helium CAP therapy significantly decreases tumor volume and inhibits tumor growth^27,60^.

The rapid growth of the glioblastoma tumor changes the function of the nearby brain tissues and leads to symptoms such as headaches, nausea, and motor and balance dysfunctions^54,61^. Motor deficits are the second most commonly reported symptoms by GBM patients which are associated with a decrease in the quality of life and the median overall survival in patients^61,62^. The results of our Ledge Beam Test showed that general motor performance significantly improved in the temozolomide and indirect argon CAP-treated groups over the course of the study. These results suggest that plasma therapy can be considered as an effective treatment for glioblastoma because it improves clinical manifestations in alignment with molecular changes.

To further investigate the possible mechanism of our CAP device on GBM in vivo, we also assessed the expression of different genes, including P53 and NLRP3. In line with our in vitro results, in vivo results also revealed that P53 gene and protein expression significantly increased in the temozolomide, direct and indirect helium, and indirect argon CAP-treated groups compared with the basal levels in the control group. The loss of p53 in human cancers contributes to aggressive tumor behavior and often promotes resistance of cancer cells to radiation and chemotherapeutic drugs^58,63^. CAP-induced apoptotic pathways in cancer cells are triggered by DNA and mitochondrial damages^50,63^ which activates several cell cycle arrest-associated proteins, including p53, as well as the expression of apoptotic signals through phosphorylation^50,53^. Therefore, the p53-dependent apoptosis following CAP therapy may be responsible for tumor suppression in our rat model of glioblastoma.

Brain malignancies are associated with various molecular pathways, pathological causes, and immunological responses^64^. It is well established that different receptors, including TLR and NLRP3, are expressed in cancerous brain tissue, and promote tumorigenesis^65^. On the other hand, some studies reported that NLRP3 inhibition reduced tumor growth and prolonged the survival rate in a mouse model of glioblastoma (70, 71). Our results revealed that NLRP3 expression significantly decreased in temozolomide, direct helium, and indirect argon CAP-treated groups compared with the baseline levels in the animal model of glioblastoma, which was in line with the increased survival rate in these groups. These results suggest that inactivation of NLRP3 can be considered a therapeutic target in brain malignancies.

It has been found that pain from a growing tumor, swallowing problems from radiation therapy, nausea, loss of appetite, or mouth ulcers, sometimes caused by chemotherapy, can lead to involuntary weight loss, which is one of the serious side effects of cancer and its treatment for many patients^66^. Hence, in order to assess the treatment side effects, animal weight (among surviving mice) was monitored throughout the study. Our observations revealed that body weight significantly decreased in the TMZ group, which is in line with a previous study by Sanghez et al.^67^ In contrast, the weight fluctuations in the groups treated with direct helium and indirect argon CAPs were not significant, indicating the non-toxicity of CAP compared to TMZ in vivo.

## Conclusion

In this study, for the first time, different plasma jets were compared on human glioblastoma tumor and normal astrocyte cells. Interestingly, the in vitro results showed that the direct application of the helium plasma jet and indirect argon plasma jet can not only induce apoptosis in these cells but also arrest their cell cycle and migration. Also, the findings indicated the very high safety of applying the argon jet indirectly to normal cells, while its effectiveness on tumor cells. In addition, the comparison of these CAPs in the glioblastoma tumor animal model showed that a single dose of direct helium and indirect argon plasma jets effectively not only reduces the size of glioblastoma tumors but also visibly improves the behavioral characteristics and locomotion and motor coordination impairments. Considering that CAP therapy showed better results on the third day and similar results compared to temozolomide on the fifth day, and taking into account the side effects of chemotherapy, CAP could hold great promise as an approach to target GBM in the future.

## Materials and methods

### Experimental procedures

This study was designed and implemented in two parts: cell line part and animal model.

### Cell line part

#### Cell culture

The human glioblastoma cancer cell line U-87 MG and the normal astrocyte CTX TNA2 cell line were purchased from the Cell Bank of Pasteur Institute (Tehran, Iran). The cells were cultured separately in complete medium composed of Dulbecco’s Modified Eagle/F-12 Media (DMEM, Life Technologies, USA) supplemented with 10% (v/v) fetal bovine serum (FBS, Sigma-Aldrich, USA), and 1% (v/v) antibiotic solution (penicillin and streptomycin, Life Technologies, USA), under standard cell culture conditions (37 ℃, 5% CO2 environment, and 95% humidity). The seeding format of the cells in the 96-well plate for the cells exposed to direct plasma was created due to the effective radius produced by the plasma flame around it, as shown in Fig. 1A.

#### Cold atmospheric plasma jet device configuration

In this research, two types of plasma jet devices were used as the Cold Atmospheric Pressure Plasma system. For helium gas, a single-electrode dielectric barrier discharge (DBD) jet was utilized, wherein the high-voltage electrode, as shown in Fig. 1B, consists of a conductive cylindrical shell placed on a dielectric insulator (quartz). On the other hand, for argon gas, the jet was in the form of a single electrode, with the difference being that the high voltage electrode was placed in the form of a needle inside a quartz tube (Fig. 1B). Both gases were powered by an AC source with a frequency of 20 kHz and a sinusoidal waveform, and the voltage of 4.5 kV. The helium and argon gas flows were set at a flow rate of 2 L per minute, controlled by a Mass Flow Meter. For the system's operation, a 2 mm diameter gas hole for open helium was used.

#### Optical emission spectroscopy measurement

Emission spectra in the 200–900 nm wavelength range of cold atmospheric helium and argon plasma jets were investigated using optical emission spectroscopy (OPTC spectroscopy, Iran). The spectrum was acquired through an optical fiber positioned at a 4 cm distance from the plasma jet nozzle.

#### Plasma treatment of cancer cells

We adopted two treatment strategies. The direct method involved directly irradiating the plasma jet to the cells in the medium (Fig. 1B). The other strategy was the indirect CAP jet, where plasma was irradiated to the culture medium without cells and then added to the cell culture (Fig. 1B). All experiments were conducted at a 4 cm distance from the emitter aperture (Based on our previous experiments whose data has not been published and the reports of past articles). Based on these two strategies, five groups were defined as follows: (a) Control group of untreated U-87 MG cells; (b) The U-87 MG cells treated with direct helium CAP; (c) The U-87 MG cells treated with indirect helium CAP; (d) The U-87 MG cells treated with direct argon CAP; (e) The U-87 MG cells treated with indirect argon CAP.

#### Cell viability assay

The cells were cultured up to passage 7. Subsequently, they were detached using 0.25% trypsin–EDTA (Life Technologies, USA) and seeded into a 96-well polystyrene microplate (Stellar Scientific) at a density of 5 × 10^3^ cells per well, with 100 µL of media. To ensure proper cell adhesion and stability, the cells were cultured for 24 h. The following day, the culture medium was removed, and a single wash with PBS eliminated dead cells. Fresh medium was introduced thereafter. To determine the IC50 dose of different CAPs, cells were subjected to CAPs treatment for 90, 120, 150, 180, and 210 s, both directly and indirectly. Subsequently, cells were incubated at 37 °C for an additional 24, 48, and 72 h post-treatment. Following this, an MTT assay was conducted to assess cell viability at 24, 48, and 72 h after treatment. The MTT (3-(4,5-Dimethylthiazol-2-Yl)-2,5-Diphenyltetrazolium Bromide) assay (Sigma-Aldrich, M2128) was performed according to the manufacturer's protocol. After rinsing the cells with PBS again, 100 μL of MTT solution was added, followed by a 3-h incubation at 37 °C in a humidified incubator with 5% CO2. After this period, the MTT solution was removed, and 100 μL of MTT solvent (0.4% HCl in anhydrous isopropanol) was gently pipetted into each well for mixing. The measurement was taken either after 10 min or within half an hour using a microplate reader (BioTek Synergy H1 Multimode Reader, Agilent, USA) at an absorbance of 570 nm, following a 30-s linear shaking. The entire set of experiments was repeated three times in triplicate for accuracy.

#### Quantification of apoptosis and cell cycle analysis by flow cytometry

After treating U-87 MG cells with CAPs IC50 doses obtained from the MTT assay, the cells were incubated for 48 h and 72 h in the helium and argon gas plasma groups, respectively. The Annexin V-FITC/PI apoptosis detection kit (Bender MedSystems, Austria) was utilized to quantify early and late apoptosis of U-87 MG cells after CAP treatment, following the manufacturer's instructions. In brief, after treatment, cells were washed in PBS, then resuspended in 100 μl of binding buffer and stained with 5 μl FITC-conjugated Annexin-V for 15 min in darkness at room temperature. Subsequently, samples were washed, resuspended in 250 μl binding buffer, and incubated with 5 μl Propidium Iodide (PI) from Sigma-Aldrich, MO, USA for 10 min. The results were analyzed using the BD FACSCalibur cytometer. Additionally, the DNA content was determined to quantify the G1, S, and G2/M phases of the cell cycle. For this purpose, following treatment, the cells were detached using 0.25% trypsin–EDTA (GIBCO, NY, USA). After detachment, the cells underwent PBS washes, followed by fixation with ice-cold 70% ethanol for 2 h. Subsequently, the cells were washed using PBS, treated with 50 μg/ml RNase A (Bio Basic, Canada) for 30 min, and then incubated with Propidium Iodide (PI; Sigma-Aldrich, USA). Flow cytometry analysis was carried out using the BD FACSCalibur cytometer.

#### Wound healing assay

Approximately 5 × 10^4^ U-87 MG cells were cultured in 24-well plates and placed inside the incubator until a confluent monolayer formed. After 24 to 48 h of incubation and the cells adhering to the bottom of the plate, a scratch was created. A sterile plastic micropipette tip was used to simulate an in vitro wound by creating a straight-edged, cell-free zone across the cell monolayer in each well. Following the scratch, the monolayer was washed with PBS, and DMEM culture medium enriched with 10% FBS was added, then returned to the incubator. The migration progress was documented by capturing sequential digital photographs of the gap using an inverted microscope (OPTIKA, Italy) at 0, 48, and 72 h after scratching. A reasonable approach is to capture three images per well per time point. The amount of cell migration was calculated by measuring the distance between the two edges of the scratch using the public domain software ImageJ, and data analysis was performed using IBM SPSS Statistics 27.0.

#### Ferric ion reducing antioxidant power (FRAP) assay

The FRAP assay is a method used to detect total antioxidant capacity (TAC). The FRAP reagent was prepared following a previously described method^68^. In brief, a mixture of 300 mM acetate buffer (pH = 3.6), 10 mM TPTZ (2,4,6-tripyridyl-s-triazine) solution in 40 mM hydrochloric acid, and 20 mM iron (III) chloride was created in a 10:1:1 ratio. To conduct the FRAP assay, 150 μL of the FRAP reagent was mixed with 20 μL of the sample, 20 μL of ascorbic acid (used as the positive control), and 20 μL of DW (used as the blank). The absorbance of each mixture was measured at 593 nm. The FRAP value was calculated using the following equation: FRAP value = [(A1 − A0)/(Ac − A0)] × 2, where Ac represents the absorbance of the positive control, A1 is the absorbance of the sample, and A0 is the absorbance of the blank.

#### Sample preparation for FRAP assay

The cultured cells were harvested from the bottom of the plates and then centrifuged at 1500 rpm at 4 °C for 5 min. The supernatant was discarded, and 200 μl of Triton X-100 was added to the cells. The mixture was pipetted and shaken for 30 min on ice. Subsequently, a sonicator device (frequency 50 Hz, amplitude 80, half cycle per second) (Ultrasonic Technology Development Company, Iran) was used to break the cells. After sonication, the mixture was centrifuged at 14,000 rpm at 4 degrees for 15 min, and the supernatant containing the cells was collected for further testing.

#### ROS estimation

To evaluate the total ROS production using a fluorescence spectrophotometer (Perkin Elmer LS-55, USA), a fresh solution of DCFDA (Sigma), was prepared in sterile Dimethyl sulfoxide (DMSO, Merck) or 100% ethanol. Subsequently, DCFDA was added to 50 mg of tissue and incubated in the dark at 37 ˚C for 45 min. To remove the cell membrane, the mixture was centrifuged at 12,000 rpm for 30 min, and the absorbance of the samples was measured at wavelengths 488 and 593 nm.

#### Immunocytochemistry

The expression of activated Caspase-3 and P53 proteins was examined using the immunocytochemistry technique. In brief, cell suspensions were cultured on sterile gelatin slides. After 24 h, the slides were washed with PBS and fixed for 20 min with 4% paraformaldehyde at 4 ˚C. Subsequently, the slides were washed again with PBS and incubated for 20 min in 2N HCl, followed by exposure to Triton X-100 for 30 min to permeabilize the cells. To block non-specific antigen sites, the slides were then incubated with 10% Goat serum for 30 min. Next, the cells were incubated overnight at 4 ˚C with primary antibodies against P53 (PA5-27822, Invitrogen) or active Caspase-3 (PA5-114687, Invitrogen) appropriately diluted with PBS (1:100). After thorough washing with PBS, the cells were exposed to secondary FITC-conjugated anti-rabbit antibodies (1:200) (31635, Invitrogen) for 1 h at room temperature in the dark. Following another round of PBS washing, DAPI was added to stain the nuclei, and the cells were examined with a fluorescent microscope (TCM 400 Binocular Microscope, Labomed). Cell counting was performed using Image j software, and the ratio of cells expressing P53 or caspase 3 to the total number of cells was reported as a percentage of P53 and caspase 3 expression.

### Animal part

#### Laboratory animals

Eight-week-old male Wistar rats weighing 200–250 g was purchased from the Pasteur Institute of Iran. The animals were housed in Plexiglas cages measuring 25 × 27 × 43 cm, under standard environmental conditions (22 ± 2 °C, humidity 45–55%, and a 12-h light/dark cycle) with free access to standard water and food.

#### GBM tumor implantation

All animal procedures were conducted in accordance with the ethical guidelines for working with animals and were approved by the ethics committee of Shahid Beheshti University of Medical Sciences (IR.SBMU.RETECH.REC.1399.025), following the principles outlined in the declaration of Helsinki. Male rats were anesthetized by intraperitoneal (i.p.) injection of a mixture containing ketamine (100 mg/kg body weight) and xylazine (10 mg/kg body weight). Afterward, they were prepared for stereotaxic surgery. A hole was created in the skull above the right hippocampus area, using specific coordinates: Anterior–Posterior (AP): 2.2 mm, Medial–Lateral (ML): 1.9 mm, Dorsal–Ventral (DV): 2 mm. Subsequently, U-87 MG cells (5 × 10^5^) in a total volume of 10 μl were injected into the right hippocampus using a Hamilton syringe. Following the injection, the Hamilton needle remained in place for 5 min before being slowly withdrawn. A sterile silicone tube with an outer width of 3 mm and a length of 1 cm was used to prepare the shunt. After the injection of cancer cells to induce the tumor, the shunt was placed at a depth of 5 mm and the wound was closed with methyl methacrylate (self-healing acrylic). In order to prevent infection, the antibiotic gentamicin was injected intramuscularly. To provide analgesia, subcutaneous buprenorphine (0.03 mg/kg) was administered 15 min before the surgery, and a meloxicam subcutaneous injection (0.2 mg/kg) was delivered immediately after the surgery. The rats were closely observed in the immediate post-operative period and excluded if any signs of abnormal neurological behavior or distress were observed. The rat model of glioblastoma was confirmed through positron emission tomography (PET) scan.

Thirty-six tumor rats were randomly divided into 6 experimental groups as follows: (a) Model group (Including six rats not treated with plasma, which underwent stereotaxic surgery and shunting procedure). (b) Direct helium CAP group (including six tumor rats treated with direct helium CAP). (c) Indirect helium CAP group (including six tumor rats that received an intracerebral injection of normal saline that was irradiated with helium plasma). (d) Direct argon CAP group (including six tumor rats exposed to direct argon CAP). (e) Indirect argon CAP group (including six tumor rats that received an intracerebral injection of normal saline that was irradiated with argon plasma). (f) Temozolomide (TMZ) group (including six tumor rats treated with temozolomide).

#### Treatment of animals with CAP and Temozolomide

After fifteen days of implanting GBM tumors in rats, plasma treatment was once administered directly (Fig. 5C-a, c) and indirectly (Fig. 5C-b) to the tumor region through a shunt embedded in the brain. The exposure time, distance of the jet from the tumor, voltage and gas flow rate of CAPs jet were selected based on the results obtained from the cell section (In-Vitro). Accordingly, an AC power supply with a frequency of 20 kHz, a sinusoidal waveform and a voltage ~ 4.5 kV was used to produce plasma, and the duration of plasma treatment was determined in seconds based on IC 50 doses. In addition, the distance between the plasma jet and the tumor was kept ~ 4 cm. Additionally, the Temozolomide drug (200 mg/m^2^)^69,70^, was administered via the tail vein to the temozolomide group, once a day, for duration of 5 days. A positron emission tomography (PET) scan was performed on three specific days: day 0, which was 15 days after tumor induction and just prior to commencing treatment with TMZ or CAPs; day 3; and day 5. Three animals were euthanized on day 5 after treatment and the other three were maintained for a duration of 8 weeks in order to participate in behavioral tests, undergo weight assessment, and have their survival status monitored.

#### Small-animal PET imaging and image analysis

The small-animal PET scans were conducted using a micro-PET scanner (Xtrim PET, Parto Negar Persia Co., Iran). Each rat received an injection of approximately 1 mCi of 18FDG via the tail vein while under general anesthesia. For every small-animal PET scan, 3-dimensional regions of interest (ROIs) were manually delineated around the tumor. Subsequently, the ROIs were converted into the tumor to background ratio (BBR) using the formula: (Brain counts per voxel) / (background counts per voxel), with the back muscles of the neck considered as the background.

#### Ledged beam test

In this test, the rats' ability to maintain balance is challenged. The test consists of 4 trials, and in each trial, the rats are required to move along a 2-m rod. If the rat successfully moves 0.5 m out of the total 2 m in each trial and manages to keep its balance, it will receive a total score of 2.

#### Immunohistochemistry

Five days after the treatment, rats were anesthetized by i.p. injection of mixed ketamine (100 mg/kg body weight) and xylazine (10 mg/kg body weight), and the tumor tissue was removed and post-fixed for 48 h at 4 °C in formalin. The fixed tissues were processed using graded alcohols and xylene and embedded in paraffin before being sectioned. Tissues were sectioned at a thickness of 5–7 μm on a microtome. The sections were deparaffinized in xylene, rehydrated with serial dilutions of alcohol, and washed with PBS in 3 steps, each lasting 5 min. To retrieve antigens, sections were incubated in 1X TBS buffer at 50–60 degrees for 20 min. Borate buffer was added for 5 min to neutralize the acid. After washing, 0.3% Triton was used for 30 min to permeabilize the cell membrane. After washing with PBS, 10% goat serum was added for 30 min to block the secondary antibody reaction. Sections were then incubated overnight at 2–8 °C with primary antibodies against GFAP (20334, Dako, Santa Clara, CA) or P53 (PA5-27822, Invitrogen). After washing with PBS, sections were incubated in secondary anti-rabbit antibody (1:2000, 7074, Cell Signaling) in the dark at 37 °C for 30 min. Then, the samples were transferred to a dark room and after washing three times, DAPI was added. After 5 min, the samples were washed with PBS and tap water. The sections were dehydrated, cleared, and covered with a coverslip. Pictures were taken using a Nikon Eclipse E600 fluorescence microscope (Japan) with a digital camera**.** Cell counting was performed using Image j software, and the ratio of cells expressing P53 or GFAP to the total number of cells was reported as a percentage of P53 and GFAP expression.

#### Gene expression analysis

Total RNA isolation was carried out using a QIAzol lysis reagent kit (Qiagen) following the manufacturer's procedure. The cDNA was produced with 500 ng total RNA using a cDNA synthesis kit (PrimeScript RT Reagent Kit; TAKARA, Japan). Primers were designed using Allele ID software (PREMIER Biosoft USA, version 7.5;) (See supplementary Table S1). The Real-time PCR was performed in a 10 mL total volume using 5 mL of RealQ Plus 2 × Master Mix Green (Amplicon, Denmark), 0.5 mL of each sense and antisense primer (10 pmol/reaction), and 25 ng cDNA. The qPCR was conducted on a Rotor-gene 6000 PCR system (Corbett Life Science) with the following program: initial denaturation at 95 °C for 15 min, followed by 40 cycles of denaturation at 95 °C for 15 s, and annealing and extension at 60 °C for 45 s. The program was followed by a melt step at 55 °C–94 °C. The relative gene expression was evaluated using the 2^-∆∆CT^ method, with the eEF1A1 gene used as the internal control.

#### Statistical data analysis

The study provides descriptive statistics as means ± standard errors. To assess the normality of the quantitative data distribution, the Shapiro–Wilk test was employed. In cases where the data distribution was found to deviate from normality, data transformation methods such as Box-Cox transformations and Inverse distribution functions^71^ were implemented. To analyze the variables among different study groups, one-way analysis of variance and Bonferroni's post hoc test were utilized. The trend in weight and the results of the ledged beam test were evaluated through repeated-measures analysis of variance, followed by post-hoc Bonferroni testing. The statistical significance level was set at *p* < 0.05, and all data analysis was performed using SPSS Statistics 27.0.

### Supplementary Information

Supplementary Information.

## Data Availability

The datasets used and/or analyzed during the current study are available from the corresponding author upon reasonable request.
