# Supplementary material for: Comparative assessment of direct and indirect cold atmospheric plasma effects, based on helium and argon, on human glioblastoma: an in vitro and in vivo study
Source: Sci Rep. 2024 Feb 13;14:3578. doi: 10.1038/s41598-024-54070-4 (PMC10861458; doi:10.1038/s41598-024-54070-4)
Supplement: Supplementary file 1 — Supplementary Information. [file 41598_2024_54070_MOESM1_ESM.pdf]

Comparative Assessment of Direct and Indirect Cold Atmospheric Plasma Effects, Based on Helium and Argon, on Human Glioblastoma: An in vitro and in vivo study

Mahdiyeh Bakhtiyari-Ramezani[1*], Mojtaba Nohekhan[1], Mohammad Esmaeil Akbari[2], Fereshteh Abbasvandi[3,2], Mahdis Bayat[3,2], Atieh Akbari[2], Meysam Nasiri[4]

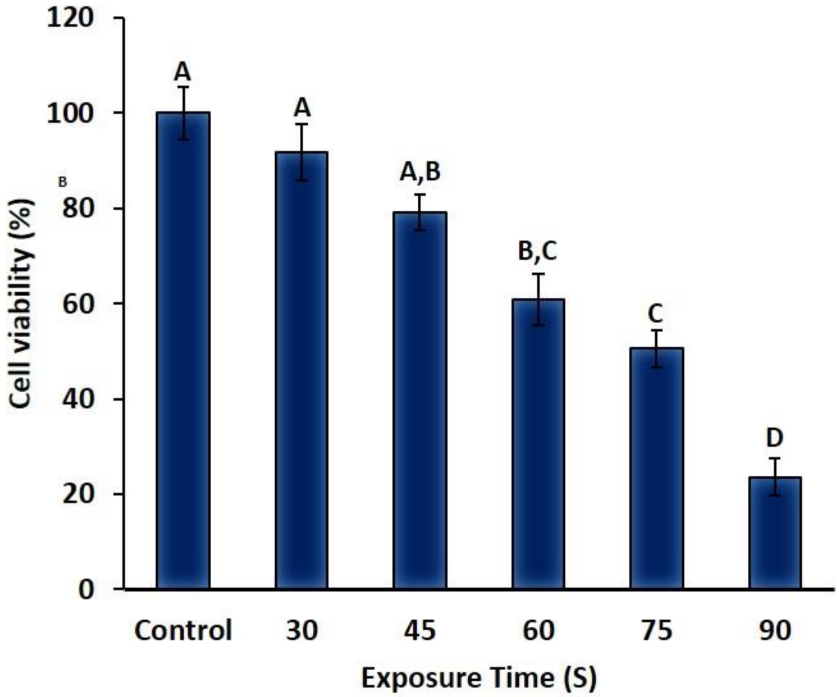

Supplementary Figure 1. Relative viability of U-87 MG cells after treatment for 30, 45, 60, 75, and 90 seconds with direct helium CAP, following 48 hours of culture after treatment with plasma. All data represent the mean ± SEM, and all experiments were performed in triplets. Data were analyzed using One-way ANOVA with Bonferroni multiple comparison post hoc test. Columns that have no letter in common with each other are meaningful ($P < 0.05$).

Supplementary Table S1. Primer sequences

Gene	*Accession number*	*Forward (5'-3')*	*Reverse (5'-3')*	*Amplicon (bp)*
TP53	NM_000546	AGGTTGGCTCTGACTGTA	GTAGATTACCACTGGAGTCTTC	123
NLRP3	NM_004895	GCCTGTTCTCATGGATTGG	CCAACCACAATCTCCGAAT	190
eEF1A1	NM_001402	TTGTTGCTGCTGGTGTTG	TCATATCTCTTCTGGCTGTAGG	160

Supplementary Table S2. The exposure time of direct and indirect cold atmospheric plasmas needed for IC50

	Cultivation time after treatments		
	24h	***48h***	***72h***
Direct Helium	98.91	70.15	34.37
Indirect Helium	256.66	137.77	152.69
Direct Argon	721.55	752.74	201.77
Indirect Argon	618.78	151.64	115.81
